# Neural Network–Based Retinal Nerve Fiber Layer Profile Compensation for Glaucoma Diagnosis in Myopia: Model Development and Validation

**DOI:** 10.2196/22664

**Published:** 2021-05-18

**Authors:** Lei Li, Haogang Zhu, Zhenyu Zhang, Liang Zhao, Liang Xu, Rahul A Jonas, David F Garway-Heath, Jost B Jonas, Ya Xing Wang

**Affiliations:** 1 State Key Laboratory of Software Development Environment School of Computer Science and Engineering Beihang University Beijing China; 2 Beijing Institute of Ophthalmology Beijing Tongren Hospital, Capital University of Medical Science Beijing Ophthalmology and Visual Sciences Key Laboratory Beijing China; 3 NIHR Biomedical Research Centre for Ophthalmology Moorfields Eye Hospital NHS Foundation Trust UCL Institute of Ophthalmology London United Kingdom; 4 Department of Ophthalmology Faculty of Medicine and University Hospital University of Cologne Cologne Germany; 5 Department of Ophthalmology Medical Faculty Mannheim Heidelberg University Mannheim Germany

**Keywords:** retinal nerve fiber layer thickness, radial basis neural network, neural network, glaucoma, optic nerve head, optical coherence tomography, myopia, optic nerve

## Abstract

**Background:**

Due to the axial elongation–associated changes in the optic nerve and retina in high myopia, traditional methods like optic disc evaluation and visual field are not able to correctly differentiate glaucomatous lesions. It has been clinically challenging to detect glaucoma in highly myopic eyes.

**Objective:**

This study aimed to develop a neural network to adjust for the dependence of the peripapillary retinal nerve fiber layer (RNFL) thickness (RNFLT) profile on age, gender, and ocular biometric parameters and to evaluate the network’s performance for glaucoma diagnosis, especially in high myopia.

**Methods:**

RNFLT with 768 points on the circumferential 3.4-mm scan was measured using spectral-domain optical coherence tomography. A fully connected network and a radial basis function network were trained for vertical (scaling) and horizontal (shift) transformation of the RNFLT profile with adjustment for age, axial length (AL), disc-fovea angle, and distance in a test group of 2223 nonglaucomatous eyes. The performance of RNFLT compensation was evaluated in an independent group of 254 glaucoma patients and 254 nonglaucomatous participants.

**Results:**

By applying the RNFL compensation algorithm, the area under the receiver operating characteristic curve for detecting glaucoma increased from 0.70 to 0.84, from 0.75 to 0.89, from 0.77 to 0.89, and from 0.78 to 0.87 for eyes in the highest 10% percentile subgroup of the AL distribution (mean 26.0, SD 0.9 mm), highest 20% percentile subgroup of the AL distribution (mean 25.3, SD 1.0 mm), highest 30% percentile subgroup of the AL distribution (mean 24.9, SD 1.0 mm), and any AL (mean 23.5, SD 1.2 mm), respectively, in comparison with unadjusted RNFLT. The difference between uncompensated and compensated RNFLT values increased with longer axial length, with enlargement of 19.8%, 18.9%, 16.2%, and 11.3% in the highest 10% percentile subgroup, highest 20% percentile subgroup, highest 30% percentile subgroup, and all eyes, respectively.

**Conclusions:**

In a population-based study sample, an algorithm-based adjustment for age, gender, and ocular biometric parameters improved the diagnostic precision of the RNFLT profile for glaucoma detection particularly in myopic and highly myopic eyes.

## Introduction

Glaucoma, as one of the most common causes of irreversible vision impairment and blindness, is diagnosed by the morphometric analysis of the optic nerve head including the peripapillary retinal nerve fiber layer (RNFL) and by psychophysical techniques such as perimetry [1-3]. These routinely applied techniques decrease in their diagnostic precision in myopic eyes and in particular, in highly myopic globes [4,5]. Due to irregularities in the refractive error and shape of the posterior part of the globe and due to high myopia-associated morphological changes in the macular region, perimetric defects lose their specificity for glaucoma and can have a multitude of causes, in addition to glaucomatous optic nerve damage [6]. Similarly, morphometric methods such as assessment of the neuroretinal rim of the optic disc and measurement of the peripapillary RNFL thickness (RNFLT) become more limited with a greater axial length of the eyes [7-11]. Furthermore, the prevalence of glaucomatous or glaucoma-like optic neuropathy increases with longer axial length, especially beyond an axial length of 26.5 mm, with odds ratios ranging from 1.6 to 3.75 for all myopic eyes and from 3.3 to 4.6 for highly myopic eyes [12-14]. These findings show the need to further improve the available methods to refine the diagnosis of glaucomatous optic neuropathy in myopic eyes.

Previous studies have shown that the thickness profile of peripapillary RNFL depends on systemic and ocular biometric parameters [15-18]. The investigations revealed that the RNFLT decreases with older age, parallel to a histomorphometrically examined loss of retinal ganglion cell axons of 0.3% per year of life, and that the peripapillary distribution of the RNFLT depends on gender, axial length, the optic disc-fovea distance, and the angle between the disc-fovea line and the horizontal (“disc-fovea angle”). In recent years, the neural network technique has been intensively studied and widely applied in computer science, including artificial intelligence in the fields of bioscience and clinical medicine [19-25]. Assuming that a neural network can transform the RNFL profile and make it comparable in eyes that differ in parameters influencing the RNFL profile, in this study, we examined whether such transformation of the RNFLT profile could improve the diagnosis of glaucoma, with special emphasis on myopic and highly myopic eyes.

## Methods

### Data Collection

Participants were randomly selected from the population-based Beijing Eye Study 2011, in which 3468 participants with an age ≥50 years were enrolled. The Medical Ethics Committee of the Beijing Tongren Hospital approved the study protocol, and all study participants gave their written informed consent. The study population and study design were described in detail previously [26,27].

Due to the relatively small number of glaucoma patients in the Beijing Eye Study, we additionally included another group of glaucoma patients who were randomly selected from the study population of the community-based Kailuan Study, which was a prospective cohort study conducted in the industrial city of Tangshan located 200 kilometers from Beijing [28]. The study was approved by the Ethics Committees of Kailuan General Hospital and followed the guidelines outlined in the Declaration of Helsinki. All participants signed a written informed consent form. Between June 2006 and October 2007, a total of 101,510 individuals (81,110 men) aged 18-98 years were recruited to participate in the study, and the participants were re-examined biannually [28]. In the re-examination period of 2014-2016, a randomly selected group of 14,400 participants from the Kailuan Study additionally underwent an ophthalmological examination including fundus photography and optical coherence tomography (OCT) of the peripapillary RNFL.

Glaucomatous optic neuropathy was defined by absolute criteria, each of which was sufficient for the diagnosis of glaucoma, and by relative criteria. The absolute criteria included a notch in the neuroretinal rim in the temporal inferior region and/or the temporal superior region, so that the inferior-superior-nasal-temporal-rule of the neuroretinal rim shape was not fulfilled; localized RNFL defects that could not be explained by any other cause than glaucoma; and an abnormally large cup in relation to the size of the optic disc. Relative criteria for the diagnosis included a markedly thinner neuroretinal rim in the inferior disc region; a diffuse decrease in the visibility of the RNFL; a marked diffuse and/or focal thinning of the retinal arteries if there was no other reason than glaucoma for retinal vessel thinning; or an optic disc hemorrhage, if there was no other reason for disc bleeding such as retinal vessel occlusions. If none of the absolute glaucoma criteria was fulfilled, the diagnosis of glaucoma required that at least 2 relative criteria had to be fulfilled, among them had to be a suspicious neuroretinal rim shape in eyes with an optic cup large enough for the assessment of the rim shape or at least 2 relative criteria had to be positive including the occurrence of an optic cup in a small optic disc, which usually would not show cupping [29]. These criteria were similar to those suggested by Foster and colleagues [30]. Using digital fundus photographs, the assessment of glaucomatous optic neuropathy was carried out by two senior graders (YXW, JBJ). In case of disagreement, the optic disc photographs were re-assessed up to 3 times, until eventually both graders agreed upon the diagnosis.

All study participants (Beijing Eye Study and Kailuan Study) underwent spectral domain OCT (Spectralis OCT; Heidelberg Engineering, Heidelberg, Germany) including a circular B-scan centered on the optic disc center with a diameter of 3.4 mm. Fundus photographs of the macula and optic disc were additionally taken (CR6-45NM Camera; Canon Inc, Ota, Tokyo, Japan). Using optical low-coherence reflectometry (Lenstar 900 Optical Biometer; Haag-Streit, Koeniz, Switzerland), biometry of the right eyes was performed for measurement of the anterior corneal curvature, central corneal thickness, anterior chamber depth, lens thickness, and axial length. The disc-fovea distance and the angle between the disc-fovea line and the horizontal (“disc-fovea angle”) were measured on fundus photographs by one grader (RAJ) [30,31]. The magnification was corrected using the Littmann-Bennett method [31,32].

We used the Heidelberg Explorer (HEE, version 5.3; Heidelberg Engineering, Heidelberg, Germany) for the automatic segmentation of the RNFL and to calculate the RNFLT. The upper border and the lower border of the RNFL were automatically outlined and generated. In rare cases with obvious misalignment, the RNFL were manually re-adjusted by trained examiners (LZ). The data of 768 RNFLT measurements equally spaced on the 360° circle were extracted, and the RNFLT profile was composed. RNFL scans with a quality score less than 15 were excluded. The data for 1 eye per individual were used for the statistical analysis.

### Training of RNFL Profile Compensation

**Figure 1:**
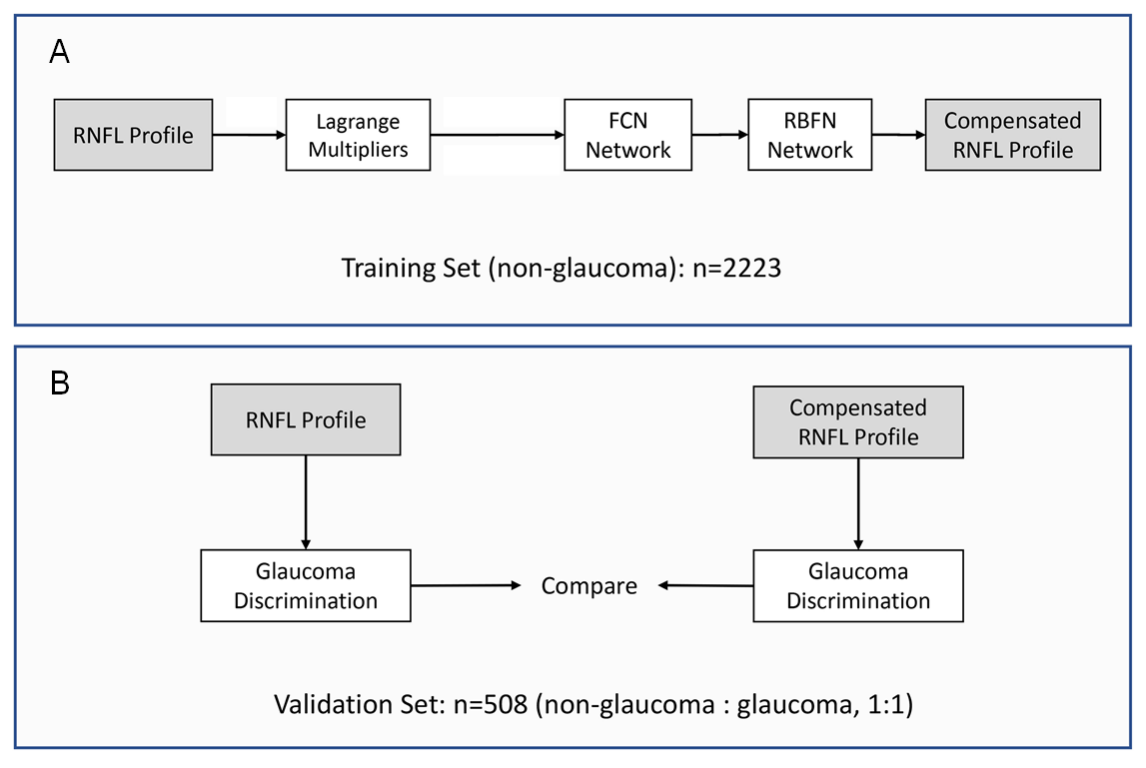
Overview of the 2-phased process in retinal nerve fiber layer (RNFL) profile compensation and its validation in discriminating glaucoma, which consisted of (A) applying the Lagrange multiplier, fully connected network (FCN), and radial basis function network (RBFN) to the training set, composed of 2223 eyes from 2223 nonglaucomatous participants, for RNFL thickness (RNFLT) compensation based on the impact of axial length (AL), age, disc-fovea angle (DFA), and disc fovea distance (DFD) and (B) evaluation of the performance of compensated RNFLT for glaucoma discrimination by comparing with the performance of the original RNFL profile.

Based on the findings obtained in previous investigations, 5 parameters shown to be associated with the RNFLT profile were chosen to be included in the present study: age, gender, axial length, the disc-fovea distance, and the disc-fovea angle. These parameters were used for the training of the RNFL profile compensation [16,31-34]. The training was performed with the images obtained from 2223 eyes from 2223 participants randomly chosen from the control group. Due to the positive correlation between older age and longer axial length, 2 independent phases were carried out. In the first phase, the parameter of age was inputted as the only factor to compensate the RNFLT vertically. Lagrange multiplier methods were applied to optimize the variance between the compensated RNFLT and the initial RNFLT, depending on the fact that each point in the RNFL profile was interassociated with neighboring points. In the second phase, the parameters of axial length, disc-fovea distance, disc-fovea angle, and gender were included in a fully connected network (FCN) for the RNFLT compensation in both the vertical and horizontal directions. The output from the FCN was further trained by a radial basis function network (RBFN) embedded with a spatial correlation, to optimize the variance between the compensated RNFLT data (Figure 1). Details of the 2-phase compensation are described in Multimedia Appendix 1.

### Validation

The validation was performed in a separate dataset containing both glaucomatous and nonglaucomatous eyes in a relationship of 1:1. The compensation algorithm was applied, and discrimination between glaucoma versus no glaucoma was carried out using either the original RNFLT profile or the compensated RNFLT profile. An eye was marked as glaucomatous if the thickness values of continuous points in the original RNFL profile or in the compensated RNFL profile were located below the single-sided 95% confidence interval of the original RNFL profile of the nonglaucomatous eyes or the compensated RNFL profile of the nonglaucomatous eyes, respectively. A receiver operating characteristic (ROC) curve including the area under the ROC curve (AUROC) was calculated to evaluate the performance of RNFLT data, in their original form and in their compensated form, for the detection of glaucoma. The accuracy, sensitivity, specificity, positive predictive value (PPV), and negative predictive value (NPV) were additionally analyzed.

## Results

**Figure 2:**
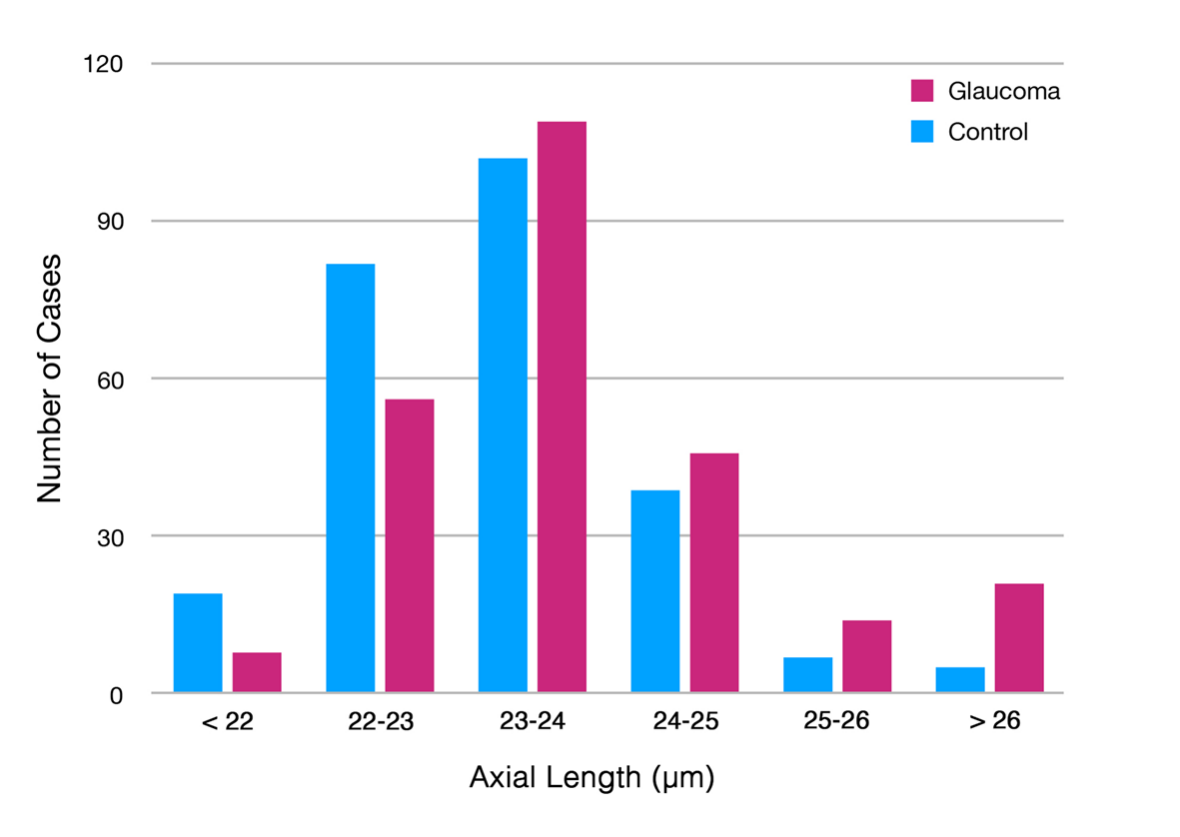
Distribution of axial length of the glaucomatous eyes and control eyes in the validation group.

Among the 3654 participants in the Beijing Eye Study 2011, 2622 eyes from 2622 participants were randomly chosen, including 2477 individuals for the control group and 145 patients with glaucoma for the study group. After adding 109 glaucomatous eyes from 109 randomly selected patients from the Kailuan Study, a total of 2731 eyes from 2731 participants (2477 control and 254 glaucoma; men: 1214/2731, 44.5%) were included, with a mean age of 63.0 (SD 9.2; range: 50-91) years. Due to an insufficient scan quality, we excluded 26 eyes (26/2731, 0.9%) from the analysis, so that the training data were eventually composed of 2223 randomly selected control eyes, and the validation group included 254 individuals in the validation control group and 254 patients with glaucoma (Table 1). The glaucomatous eyes had a longer axial length (mean 23.77, SD 1.28 mm) as compared with the nonglaucomatous eyes (mean 23.30, SD 0.96 mm) in the validation set (*P*<.001; Figure 2).

**Table 1:** Demographic and ocular parameters of the study population.

Eye sets		Age (years), mean (SD, range)	Gender (male), n (%)		Axial length (mm), mean (SD, range)	Disc-fovea distance (mm), mean (SD, range)	Disc-fovea angle (°), mean (SD, range)	Mean RNFLT^a^ (µm), mean (SD, range)
All (n=2731)		63.0 (9.2, 50 to 91)		1214 (44.5)	23.23 (1.01, 18.96 to 28.87)	4.93 (0.39, 3.68 to 7.63)	7.64 (3.51, –16.64 to 23.25)	101 (12, 32 to 147)
Normal (n=2477)		62.3 (8.9, 50 to 91)	1076 (43.4)		23.18 (0.96, 18.96 to 28.87)	4.88 (0.27, 3.68 to 5.99)	7.59 (3.4, –16.64 to 23.25)	102 (11, 43 to 147)
Glaucoma (n=254)		69.4 (9.3, 50 to 90)	138 (54.3)		23.77 (1.27, 19.59 to 28.84)	5.41 (0.82, 3.8 to 7.63)	8.16 (4.36, –13.14 to 22.59)	85 (17, 33 to 122)
**Training set**								
	All eyes (n=2223)	62.2 (8.9, 50 to 91)	960 (43.2)		23.16 (0.96, 18.96 to 28.87)	4.88 (0.27, 3.68 to 5.99)	7.67 (3.42, –16.64 to 23.25)	102 (11, 43 to 141)
	10% longest eyes (n=222)	63.5 (8.7, 50 to 85)	145 (65.3)		25.08 (0.77, 24.32 to 28.78)	4.86 (0.3, 3.86 to 5.99)	7.49 (3.86, –6.3 to 23.25)	96 (10, 60 to 119)
	20% longest eyes (n=444)	63.41 (8.77, 50 to 85)	290 (65.3)		24.56 (0.76, 23.83 to 28.87)	4.83 (0.28, 3.68 to 5.99)	7.64 (3.55, –6.3 to 23.25)	98 (11, 43 to 124)
	30% longest eyes (n=666)	63.3 (8.97, 50 to 90)	408 (61.2)		24.26 (0.75, 23.53 to 28.87)	4.84 (0.27, 3.68 to 5.99)	7.62 (3.4, –6.3 to 23.25)	100 (11, 43 to 139)
**Validation set**								
	All eyes n=508)	66.4 (9.6, 50 to 90)	254 (50. 0)		23.53 (1.15, 19.59 to 28.84)	5.14 (0.67, 3.8 to 7.63)	7.54 (3.87, –13.14 to 22.59)	94 (17, 32 to 147)
	Glaucoma in all eyes (n=254)	69.4 (9.3, 50 to 90)	138 (54.3)		23.77 (1.28, 19.59 to 28.84)	5.41 (0.82, 3.8 to 7.63)	8.16 (4.37, –13.14 to 22.59)	86 (17, 32 to 122)
	10% longest eyes (n=51)	68.3 (9.3, 50 to 90)	32 (62.7)		26.01 (0.89, 24.95 to 28.84)	5.3 (0.9, 4.05 to 7.63)	8.44 (4.39, –2.05 to 22.59)	82 (16, 40 to 122)
	Glaucoma in 10% longest eyes (n=37)	68.78 (9.8, 50 to 90)	23 (62.2)		26.2 (0.93, 24.95 to 28.84)	5.46 (0.99, 4.05 to 7.63)	8.88 (4.92, –2.05 to 22.59)	80 (17, 40 to 122)
	20% longest eyes (n=102)	67.8 (9.2, 50 to 90)	66 (64.7)		25.26 (0.99, 24.21 to 28.84)	5.34 (0.85, (4.05 to 7.63)	7.92 (3.99, –2.05 to 22.59)	88 (17, 38 to 122)
	Glaucoma in 20% longest eyes (n=66)	68.1 (9.2, 50 to 90)	41 (62.1)		25.44 (1.08, 24.21 to 28.84)	5.58 (0.95, 4.05 to 7.63)	8.5 (4.43, –2.05 to 22.59)	83 (17, 38 to 122)
	30% longest eyes (n=153)	67.2 (9.0, 50 to 90)	99 (64.7)		24.86 (0.99, 23.92 to 28.84)	5.27 (0.77, 3.8 to 7.63)	7.51 (4.18 (–13.14 to 22.59)	90 (17, 38 to 147)
	Glaucoma in 30% longest eyes (n=91)	67.8 (9.0, 50 to 90)	57 (62.6)		25.06 (1.11, 23.92 to 28.84)	5.5 (0.9, 3.8 to 7.63)	7.91 (4.79, –13.14 to 22.59)	85 (17, 38 to 122)

^a^RNFLT: retinal nerve fiber layer thickness.

**Figure 3:**
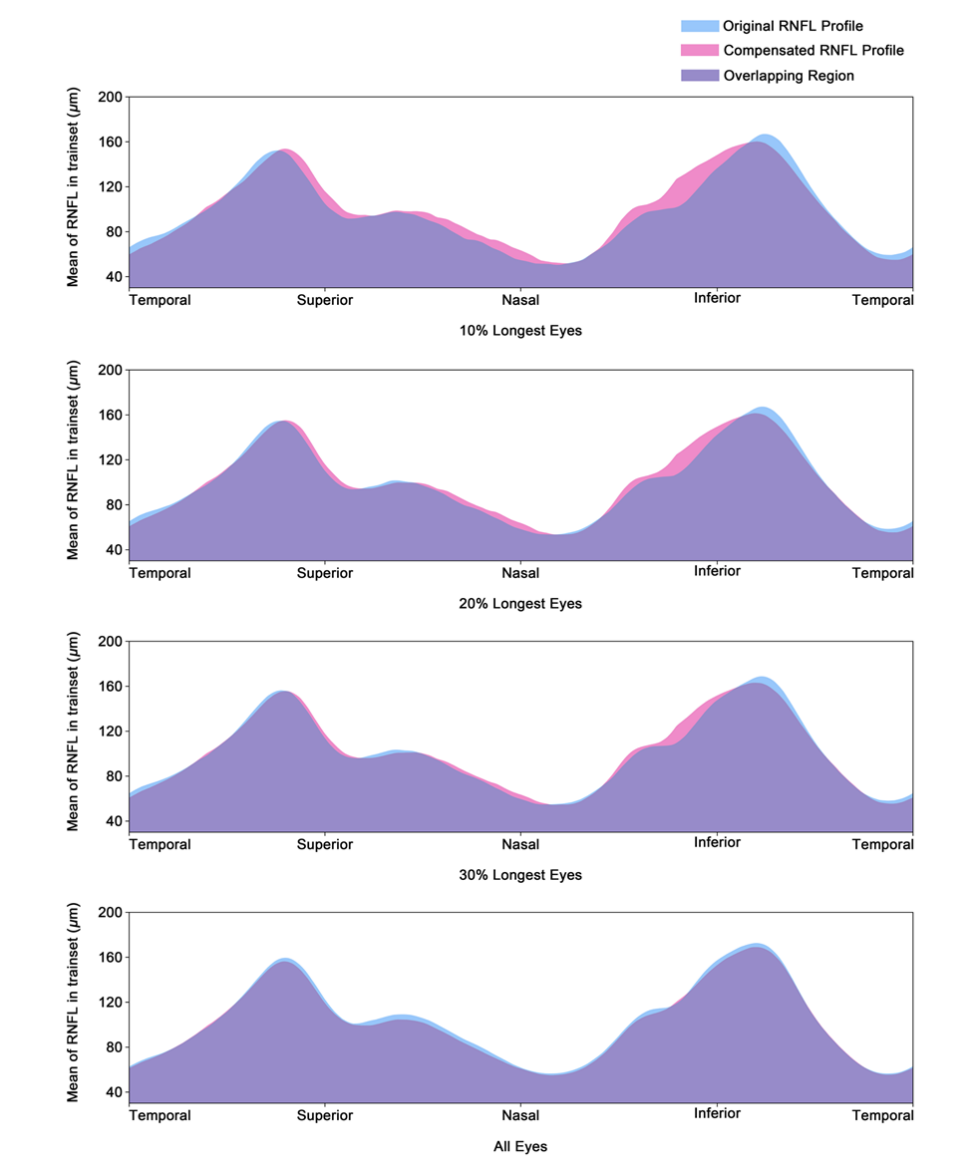
The mean original retinal nerve fiber layer (RNFL) profile (blue) and the mean compensated RNFL profile (pink) of the 10% longest eyes, 20% longest eyes, 30% longest eyes, and all eyes.

The compensation-induced change in the RNFLT values in height (vertical) and in location (horizontal) increased with longer axial length (Figure 3). It was most marked in the eyes with the longest axial length: The subgroup of eyes in the highest 10% percentile of the axial length distribution (mean axial length 25.08, SD 0.77 mm) had the highest compensation, followed by the subgroup of eyes in the highest 20% percentile of the axial length distribution (mean 24.56, SD 0.76 mm) and the subgroup of eyes in the highest 30% percentile of the axial length distribution (mean 24.26, SD 0.75 mm). The mean difference between the uncompensated RNFLT values and the compensated values was negligible in the eyes with an axial length outside of the 30% percentile of the longest axial length (mean axial length 23.16, SD 0.96 mm; Figure 3).

**Figure 4:**
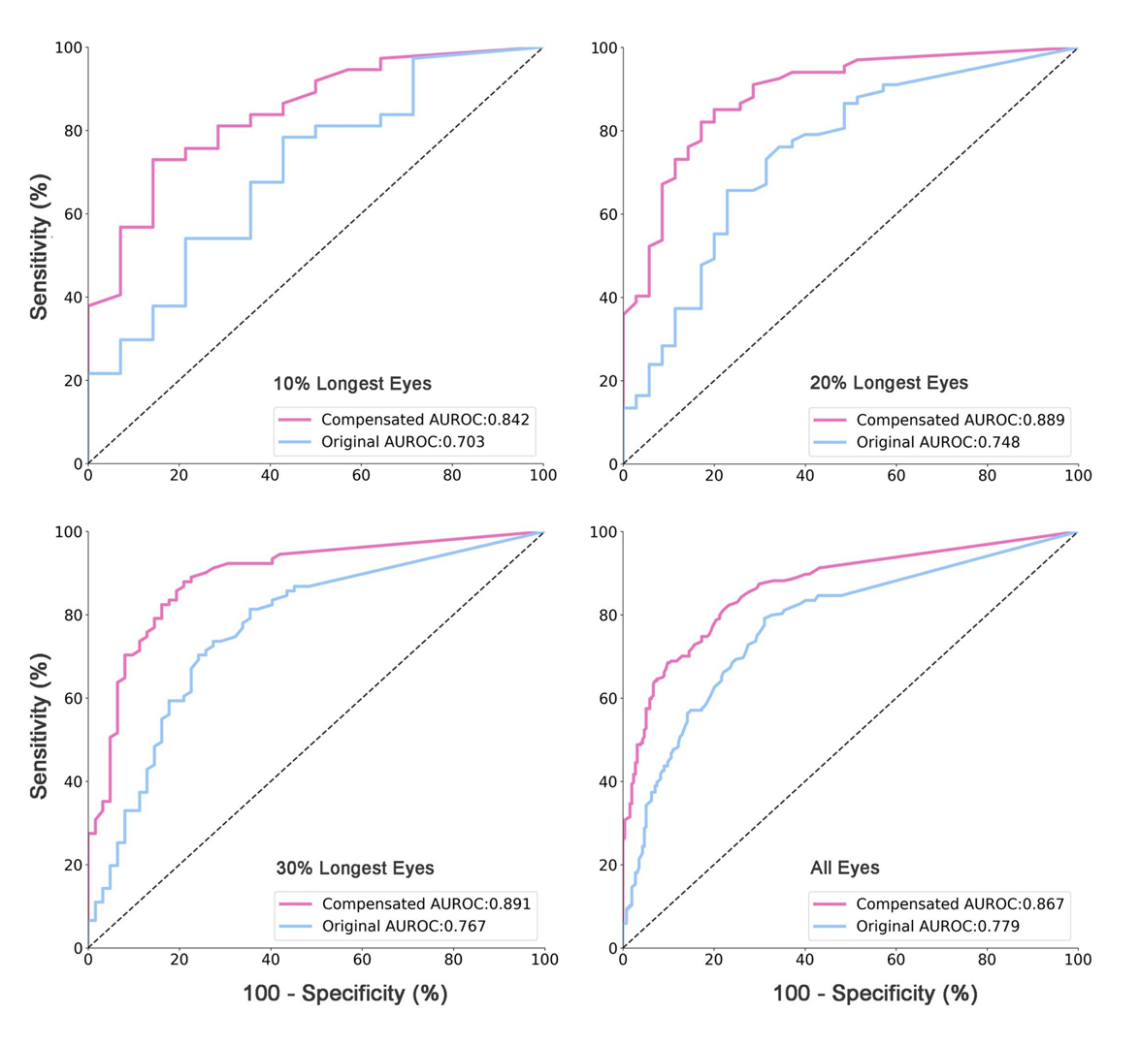
Area under receiver operation curve (AUROC) for the detection of glaucoma in the validation data set before (blue line) and after (pink line) the transformation, in eyes of the 10% longest axial length (mean 26.01 mm), 20% longest axial length (mean 25.26 mm), 30% longest axial length (mean 24.86 mm), and all eyes (mean 23.53 mm).

Comparing the compensated RNFLT values with the uncompensated RNFLT values revealed that the AUROC for the detection of glaucoma increased from 0.70 to 0.84, from 0.75 to 0.89, from 0.77 to 0.89, and from 0.78 to 0.87, for eyes within the 10% highest length percentile, eyes within the 20% highest length percentile, eyes within the 30% highest axial length percentile, and all eyes, respectively (Figure 4). The relative increase was more pronounced in eyes with longer axial length, with an increase by 19.8%, 18.9%, 16.2%, and 11.3% in the highest 10% percentile subgroup, highest 20% percentile subgroup, highest 30% percentile subgroup, and all eyes, respectively. The accuracy, sensitivity, specificity, PPV, and NPV of the original and compensated RNFL in subgroups are shown in Table 2.

**Table 2:** Performance of the original retinal nerve fiber layer (RNFL) and the compensated RNFL to detect glaucoma in subgroups and all eyes of the validating dataset.

Eye groups		Accuracy	Sensitivity	Specificity	Positive predictive value	Negative predictive value
**10% longest eyes**						
	Original	0.073	0.784	0.571	0.829	0.500
	Compensated	0.077	0.730	0.857	0.931	0.545
**20% longest eyes**						
	Original	0.140	0.657	0.771	0.846	0.540
	Compensated	0.165	0.821	0.829	0.902	0.707
**30% longest eyes**						
	Original	0.220	0.736	0.726	0.798	0.652
	Compensated	0.250	0.824	0.839	0.882	0.765
**All eyes**						
	Original	0.740	0.791	0.689	0.718	0.768
	Compensated	0.795	0.811	0.779	0.786	0.805

## Discussion

### Principal Findings

In our population-based study, the diagnostic precision of the peripapillary RNFLT profile for the detection of glaucoma increased when the dependence of the RNFLT profile on age and the ocular biometric parameters of axial length, disc-fovea distance, and disc-fovea angle were taken into account by applying 2 neural networks. These networks, FCN and RBFN, developed an algorithm by which the RNFLT profile was transformed either horizontally or vertically. Applying the algorithm increased the diagnostic performance of the RNFLT profile, which was markedly better with longer axial length. The improvement in relative percentage points as measured by the AUROC was 19.8% in the subgroup of eyes within the highest 10% percentile group, 16.2% in the highest 30% percentile subgroup, and 11.3% in all eyes of the study population.

Myopia-related changes in the appearance of the optic nerve head can make the detection of additional changes caused by glaucomatous optic neuropathy in myopic eyes difficult [5]. The parapapillary gamma zone and delta zone in myopic eyes increase the brightness of the background so that the visibility of the retinal nerve fiber layer upon ophthalmoscopy is reduced due to a physical-optical effect. The presence of a gamma and delta zone additionally leads to irregularities in the profile of the tissues underlying the RNFL, so that the automatic delineation of the inner retinal layer containing the retinal nerve fibers from the subsequent layer gets more difficult. The axial elongation–associated increase in the parapapillary region by the development of the gamma and delta zone can lead to a thinning of the RNFL due to geometric reasons. In moderate myopia, the Bruch’s membrane opening as the inner opening layer of the optic nerve head usually shifts temporally in the direction of the fovea, leading to an overhanging of the Bruch’s membrane at the nasal optic disc border and a lack of the Bruch’s membrane at the temporal disc border (ie, gamma zone) [35]. The resulting oblique course of the retinal ganglion cell axons through the myopic optic nerve head canal as compared to a perpendicular course in emmetropic eyes leads to a change in the configuration of the neuroretinal rim in myopic eyes, rendering the detection of glaucomatous rim changes more difficult. The axial elongation–associated enlargement of the optic disc is associated with a stretching of the lamina cribrosa so that the depth of the optic cup may be reduced. It leads to decreased spatial contrast between the height of the neuroretinal rim and the depth of the optic cup and thus renders the delineation of the rim from the cup more difficult. Simultaneously, the color of the rim changes from pink in direction to yellow, so that the color contrast between the rim and optic cup decreases in myopic eyes, again rendering the differentiation of the rim from the optic cup more difficult. As also pointed out earlier in the paragraph, perimetric changes also lose their specificity for glaucomatous optic nerve damage as their cause. The axial elongation–associated changes can also present with perimetric defects that mimic or cover a glaucoma-related visual field defect. These changes might include diffuse peripapillary and macular chorioretinal atrophy, macular Bruch’s membrane defects, and scleral staphylomas. Furthermore, the intraocular pressure in myopic eyes with glaucomatous can be within the normal range since the myopia-associated stretching and thinning of the lamina cribrosa and peripapillary scleral flange may increase the pressure susceptibility of the optic nerve fibers when passing through the lamina cribrosa. These examples may demonstrate the need for improved morphometric glaucoma diagnosis in myopic eyes [4,5].

Previous studies showed that the thickness profile of the RNFL depended on other morphologic parameters such as axial length, the disc-fovea distance, and the disc-fovea angle [31,32]. The longer the axial length and disc-fovea distance were, the smaller the angle kappa between the temporal superior and temporal inferior vascular arcade, which accompanies the RNFL branches. The disc-fovea angle was a surrogate for sagittal rotation of the optic nerve head, also influencing the location of the RNFLT profile. By taking these associations of the RNFLT profile into consideration and adjusting for them using a compensation algorithm, there was an improvement in the diagnostic precision of the RNFLT profile for the detection of glaucoma (Figures 3 and 4). The improvement was more marked with more myopic eyes.

The AUROC values found in our study population are roughly comparable to those of previous investigations. To cite examples, Shoji and colleagues [9,35] examined 31 patients with high myopia and 51 patients with high myopia and glaucoma and found that the peripapillary RNFLT had an AUROC of 0.83 in the discrimination of normal eyes from glaucomatous eyes. Kim and associates [36] reported that the ability to detect glaucomatous changes in a highly myopic group (n=45) by RNFL examination had an AUROC of 0.83. When comparing the various studies, one may consider that they markedly differed in the size and composition of their study population. In particular, our study population was recruited in a population-based manner. Subsequently, the glaucoma patients showed all stages, including early stages, of glaucomatous optic neuropathy. In addition, the nonglaucomatous group in our study population included eyes with nonglaucomatous optic nerve damage in addition to other pathological conditions like retinal diseases, nonglaucomatous neuropathies, and cataract. If we had included only eyes without any (nonglaucomatous) optic nerve damage and without any retinal disease in the control group, separating the glaucomatous study and control group would have been easier, and the AUROC would have been higher.

The findings that the height and profile of the peripapillary RNFLT were associated with various ocular and systemic parameters were also found in other investigations. Yamashita and colleagues [37] noted that the position of the superior-temporal RNFLT peak was associated with the location of the papillomacular position, optic disc tilt, and body height, while the inferior-temporal RNFL peak position was correlated with corneal thickness and axial length. Leung et al [8] investigated 189 myopic eyes and reported that the angle between the superotemporal and inferotemporal RNFL bundles decreased with longer axial length. Fujino et al [38] found that a RNFLT profile correction based on the retinal vessel position in all twelve 30° sectors was able to improve the structure-function relationship in all sectors. Rho et al [39] adjusted the 1% reference line of the RNFLT profile according to the retinal vessel position, by which they obtained better agreement with the standard diagnosis of glaucoma. These previous studies on the dependence of the RNFLT profile on other ocular parameters revealed, however, that these associations with the RNFLT profile change were not linear and that the effect of a correction by a linear mathematical method was limited. In this study, the FCN and RBFN were used to compensate the RNFLT profile in both the horizontal direction (position shift) and vertical direction (thickness change). Decreasing the systemic variability of the RNFLT profile resulted in an improvement of the diagnostic performance for glaucoma detection, especially in highly myopic patients.

### Limitations

When discussing the results of our study, its limitations should be taken into account. First, compensation of the RNFLT profile was based on data from participants with an age ≥50 years, and the performance of glaucoma detection was not validated in younger participants. Second, the composition of the validation dataset included a 1:1 ratio of glaucomatous eyes to nonglaucomatous eyes. However, since the prevalence of glaucoma increases with axial length, a relatively high proportion of glaucomatous eyes in the validation set may reflect the higher prevalence of glaucoma in eyes with myopia and high myopia. The strengths of the study included that the large population-based dataset offered an opportunity to observe the diverse patterns of the nonlinear relationship between the RNFLT profile and axial length. An RBFN with the advantages of good generalization, strong tolerance to input noise, and online learning ability made it possible to interpret the patterns to a reliable compensation. Due to the population-based recruitment of the study population, the validation group included glaucomatous eyes of all glaucoma stages, so that the results are more generalizable than in hospital-based studies with a preponderance of advanced glaucoma stages in the study groups.

### Conclusion

Applying an algorithm to adjust the nonlinear dependence of the RNFLT profile on age, axial length, disc-fovea distance, and disc-fovea angle resulted in improved diagnostic precision of the peripapillary RNFLT profile for the detection of glaucoma in a population-based study population. The improvement in the diagnostic precision of the compensated versus uncompensated RNFLT profile data increased in relative terms with longer axial length. With an increase of 20%, it was most marked in the highly myopic group. The application of this neural network–based RNFLT profile compensation may also be helpful to improve glaucoma diagnosis in myopic eyes in clinical practice.

## Appendix

Multimedia Appendix 1 Details of the two-phase-compensation of retinal nerve fiber layer.

## Abbreviations

AUROC: area under the receiver operating characteristic curve
FCN: fully connected network
NPV: negative predictive value
OCT: optical coherence tomography
PPV: positive predictive value
RBFN: radial basis function network
RNFL: retinal nerve fiber layer
RNFLT: retinal nerve fiber layer thickness
ROC: receiver operating characteristic
