# Supplementary material for: Neural Network–Based Retinal Nerve Fiber Layer Profile Compensation for Glaucoma Diagnosis in Myopia: Model Development and Validation
Source: JMIR Med Inform. 2021 May 18;9(5):e22664. doi: 10.2196/22664 (PMC8170554; doi:10.2196/22664)
Supplement: Multimedia Appendix 1 [file medinform_v9i5e22664_app1.pdf]

Supplement: Details of the Two-Phase-Compensation of Retinal Nerve Fiber Layer

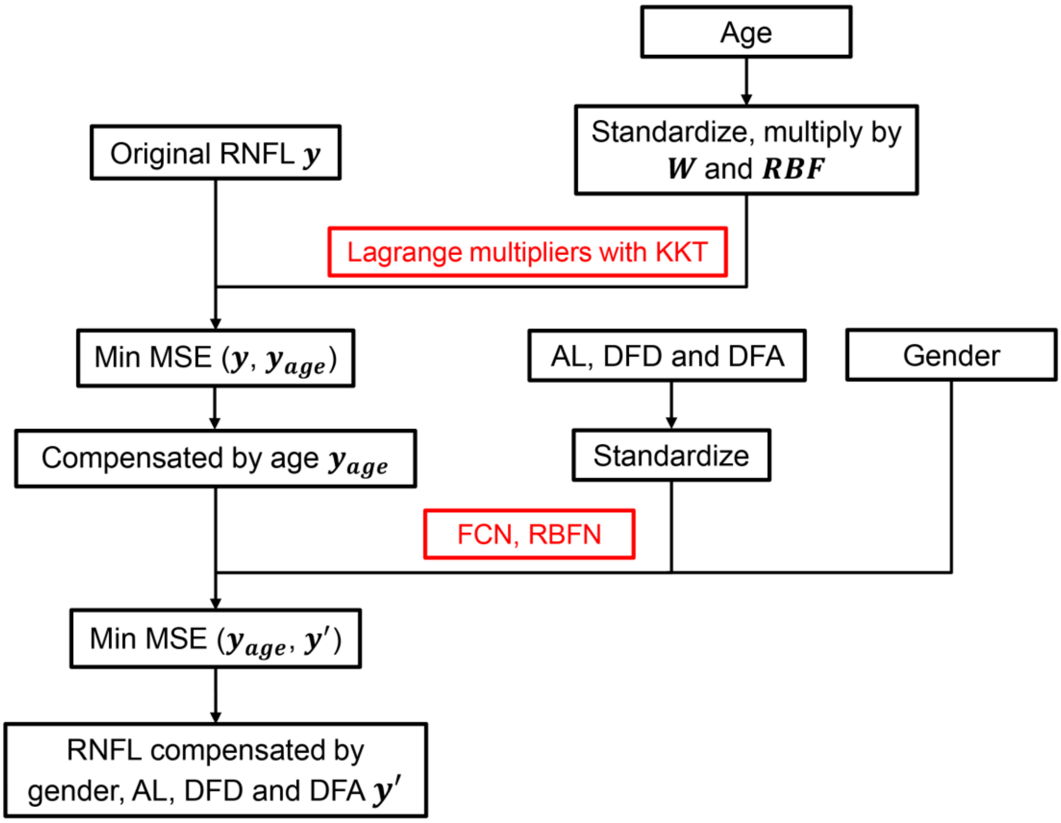

Figure 1. Diagrams showing an overview of the method.

As shown in Figure 1, the method of compensating retinal nerve fiber layer (RNFL) consists of two phases. In the first phase, the age is used to compensate RNFL solely in vertical direction. The compensated RNFL by age is used in the second phase. In the second phase, gender, axial length (AL), disc-fovea angle (DFA) and disc-fovea distance (DFD) are used to compensate RNFL in vertical direction and horizontal direction concurrently.

Phase 1. Compensate RNFL by age

Age is represented by the one-dimensional vector $\boldsymbol{x}$ and standardized. And the bias vector with all elements are 1 is superadded to $\boldsymbol{x}$. RNFL profile is represented by the *N*-dimensional vector $\boldsymbol{y}$ with $N = 768$. The compensated RNFL by age $\boldsymbol{y}_{age}$ is represented by formula (1):

$$\boldsymbol{y}_{age} = [\boldsymbol{x}_{standard} \quad 1] \times \boldsymbol{W}_{2\times M} \times \boldsymbol{RBF}_{M\times N} + \boldsymbol{y}_{train} \tag{1}$$

The product $[\boldsymbol{x}_{standard} \quad 1] \times \boldsymbol{W}_{2\times M} \times \boldsymbol{RBF}_{M\times N}$ is the movement in vertical direction. $\boldsymbol{W}_{2\times M}$ is the weight matrix and its elements are initialized randomly. The element at position (m, n) of matrix $\boldsymbol{RBF}_{M\times N}$ is defined by formula (2):

$$RBF_{(m,n)} = \varphi(m, q) = exp\{\frac{-||m,q||^2}{2\sigma^2}\} \quad (2)$$

$m = \{1,2,\dots,M\}$. $n = \{1,2,\dots,N\}$. $||m,q||$ is the Euclidean distance between m and q. $q = \frac{N}{M-1} \times m \bmod N$ indicates that it is equally spaced on the scanned circle of RNFL. $\sigma = \lfloor \frac{N}{M-1} \rfloor$.

According to the fact that the RNFL thickness (RNFLT) decrease with ageing, we consider to use the optimization algorithm with constraint conditions to work out the weight matrix $\boldsymbol{W}_{2\times M}$. Generally we use Mean Squared Error (MSE) between $\boldsymbol{y_{age}}$ and the mean of $\boldsymbol{y_{train}}$ as objective function. To minimize the objective function, Lagrange multipliers with Karush-Kuhn-Tucker conditions are used. Then the weight matrix $\boldsymbol{W}_{2\times M}$ can be worked out and $\boldsymbol{y_{age}}$ can be acquired by formula (1) and $\boldsymbol{y_{age}}$ will be used in phase 2.

Phase 2. Compensate RNFL by gender, AL, DFA and DFD

Transformation of RNFLT Profile

Approximation was applied in the establishment of RNFL profile by integrating N discrete points with $N-1$ straight lines. The approximation was furtherly described as the following formula (3):

$$\boldsymbol{g}(\boldsymbol{y},\boldsymbol{p}) = \boldsymbol{y}_{\lfloor \boldsymbol{p} \rfloor \bmod N} + (\boldsymbol{p} - \lfloor \boldsymbol{p} \rfloor) \times (\boldsymbol{y}_{\lfloor \boldsymbol{p}+1 \rfloor \bmod N} - \boldsymbol{y}_{\lfloor \boldsymbol{p} \rfloor \bmod N}) \quad (3)$$

$\boldsymbol{g}(\boldsymbol{y},\boldsymbol{p})$ represents the thickness at positions $\boldsymbol{p}$ on the RNFLT curve derived from $\boldsymbol{y}$. $\boldsymbol{y_p}$ represents the elements of $\boldsymbol{y}$ at $\boldsymbol{p}$ positions. $\boldsymbol{p}$ is the *N*-dimensional vector and its elements represent new positions after compensating in horizontal direction. We define $\boldsymbol{p} = \boldsymbol{i} + \boldsymbol{\Delta p}$ and $\boldsymbol{i} = [0,1,\dots,N-1]$ is an *N*-dimensional vector representing initial positions. $\boldsymbol{\Delta p}$ is an *N*-dimensional vector that represents the compensation of positions in horizontal direction. Along with the *N*-dimensional vector $\boldsymbol{s}$ representing the movements in vertical direction, we define the compensated RNFL curve by formula (4):

$$\boldsymbol{y}' = \boldsymbol{s} \times \boldsymbol{g}(\boldsymbol{y},\boldsymbol{p}) \quad (4)$$

Estimations of $\boldsymbol{s}$ and $\boldsymbol{p}$

Depending on formula (4), we need work out $\boldsymbol{s}$ and $\boldsymbol{p}$ to obtain the compensated RNFL $\boldsymbol{y}'$. Anatomically, each point in the RNFL profile is in related with neighboring points. Thus a spatial correlation can be embedded in the radial basis function network (RBFN), with which the fully connected network (FCN) was combined. The estimation of $\boldsymbol{s}$ is similar to the process of estimating $\boldsymbol{\Delta p}$. So, in the following parts we will first introduce some key components in the RBF neural network to predict $\boldsymbol{\Delta p}$.

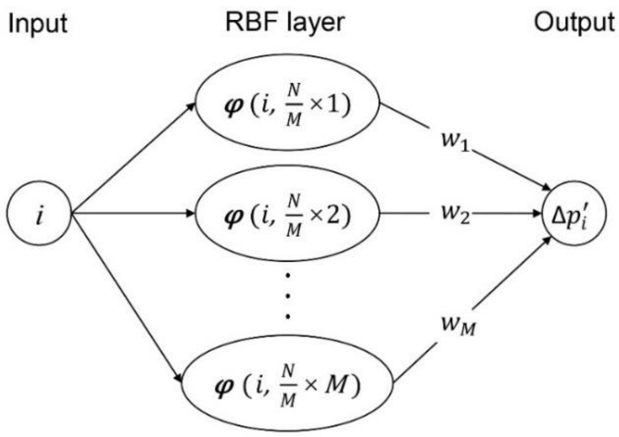

Figure 2. The structure of RBFN. The input is the position i and the output is $\Delta p_i'$. The activation function is the radial basis function.

As shown in Figure 2, the input of RBFN is a position i that is an element of vector $\boldsymbol{i}$ and the output $\Delta p_i'$ defined by formula (5) represents the horizontal compensation at the position i. The activation function is the radial basis function defined by formula (2).

$$\Delta p_i' = \sum_{j=1}^{M} w_j\, \boldsymbol{\varphi}\,(i, \frac{N}{M} \times m) \tag{5}$$

w_j is an element of the *M*-dimensional vector $\boldsymbol{w}$ that is the weight vector in RBFN. For estimating $\Delta p_i'$ we must calculate the weight vector $\boldsymbol{w}$ in RBFN. Heuristically, the three factors AL, DFA and DFD are composed as 3-dimensional vector $\boldsymbol{x\_fc}$ and standardized. The factor gender is a bivariate variable so it is not standardized. The gender and the standardized AL, DFA and DFD are composed as 4-dimensional vector $\boldsymbol{x\_fc}$ are passed to the FCN with 4 input features and M output features. It implies that there is a weight matrix $\boldsymbol{W\_FC}_{4\times M}$ in FCN and it can be worked out by training the FCN. Then we can calculate weight vector $\boldsymbol{w}$ in RBFN by formula (6):

$$\boldsymbol{w} = \boldsymbol{x\_fc} \times \boldsymbol{W\_FC}_{4\times M} \tag{6}$$

Subsequently, we can get vector $\boldsymbol{\Delta p'}$ consisting of the elements $\Delta p_i'$. We constrain the horizontal compensation by a constant L and a function $\boldsymbol{tanh}(x) = \frac{e^x - e^{-x}}{e^x + e^{-x}}$, i.e., $\boldsymbol{\Delta p} = L \times \boldsymbol{tanh}(\boldsymbol{\Delta p'})$. Then $\boldsymbol{p}$ can be worked out by $\boldsymbol{p} = \boldsymbol{i} + \boldsymbol{\Delta p}$.

The estimation of vertical compensation $\boldsymbol{s}$ is same as the process of estimating $\boldsymbol{\Delta p}$. But the estimation of $\boldsymbol{s}$ has different weight matrix such as $\boldsymbol{W\_FC}$ in FCN. The different weight matrix in FCN for $\boldsymbol{s}$ and $\boldsymbol{p}$ can be worked out by training the FCN concurrently. The loss function in FCN is MSE between $\boldsymbol{y'}$ and the mean of $\boldsymbol{y_{age}}$ with L2 regularization. Furthermore, $\boldsymbol{y}$ in formula (3) and (4) must be replaced by $\boldsymbol{y_{age}}$. So far, $\boldsymbol{s}$ and $\boldsymbol{p}$ have been worked out. Using $\boldsymbol{y_{age}}$, $\boldsymbol{s}$ and $\boldsymbol{p}$, the compensated RNFL $\boldsymbol{y'}$ can be acquired by formula (3) and (4).
